# Revealing the chemical separated two-phase structure in lithium-manganese-rich cathode

**DOI:** 10.1093/nsr/nwaf202

**Published:** 2025-05-21

**Authors:** Jiayi Wang, Xincheng Lei, Hao Meng, Pengxiang Ji, Tenglong Lu, Weijun Liang, Xiaozhi Liu, Sheng Meng, Lin Gu, Miao Liu, Xin Wang, Dong Su

**Affiliations:** Institute of Carbon Neutrality, Zhejiang Wanli University, Ningbo 315100, China; Beijing National Laboratory for Condensed Matter Physics, Institute of Physics, Chinese Academy of Sciences, Beijing 100190, China; School of Physical Sciences, University of Chinese Academy of Sciences, Beijing 100049, China; Institute of Carbon Neutrality, Zhejiang Wanli University, Ningbo 315100, China; Beijing National Laboratory for Condensed Matter Physics, Institute of Physics, Chinese Academy of Sciences, Beijing 100190, China; School of Physical Sciences, University of Chinese Academy of Sciences, Beijing 100049, China; Beijing National Laboratory for Condensed Matter Physics, Institute of Physics, Chinese Academy of Sciences, Beijing 100190, China; Songshan Lake Materials Laboratory, Dongguan 523808, China; Beijing National Laboratory for Condensed Matter Physics, Institute of Physics, Chinese Academy of Sciences, Beijing 100190, China; Beijing National Laboratory for Condensed Matter Physics, Institute of Physics, Chinese Academy of Sciences, Beijing 100190, China; School of Physical Sciences, University of Chinese Academy of Sciences, Beijing 100049, China; Songshan Lake Materials Laboratory, Dongguan 523808, China; Beijing National Center for Electron Microscopy and Laboratory of Advanced Materials, Department of Materials Science and Engineering, Tsinghua University, Beijing 100084, China; Beijing National Laboratory for Condensed Matter Physics, Institute of Physics, Chinese Academy of Sciences, Beijing 100190, China; Songshan Lake Materials Laboratory, Dongguan 523808, China; Center of Materials Science and Optoelectronics Engineering, University of Chinese Academy of Sciences, Beijing 100049, China; Institute of Carbon Neutrality, Zhejiang Wanli University, Ningbo 315100, China; Beijing National Laboratory for Condensed Matter Physics, Institute of Physics, Chinese Academy of Sciences, Beijing 100190, China; School of Physical Sciences, University of Chinese Academy of Sciences, Beijing 100049, China

**Keywords:** Li-ion battery, lithium-manganese-rich cathode, phase separation, solid solution, ion migration, transmission electron microscopy

## Abstract

Lithium-manganese-rich (LMR) oxides are regarded as one of the most promising cathode materials for next-generation batteries. However, their poor rate capability and performance degradation during cycling present significant challenges for practical applications. Understanding how to optimize their microscopic structures during synthesis may provide critical insights for enhancing their performance. In this work, we investigated the structural evolution during the solid-state sintering of Li_1.2_Ni_0.2_Mn_0.6_O_2_ from Li-/Mn-/Ni-carbonate precursors. Combining X-ray diffraction and transmission electron microscopy (TEM) techniques, we observed the nucleation of a nanoscaled solid-solution phase at 550°C, accompanied by secondary phases of spinel-like, layered and rock salt. At 800°C, a relatively pure solid-solution phase *R3̅m* is formed. Notably, we uncovered, for the first time, a phase transition from a solid-solution structure to a chemically separated two-phase structure when annealing the sample from 850°C to 900°C. Atomic resolution scanning-TEM (STEM) imaging clearly distinguished the *C2/m* phase from the *R3̅m* phase, separated by a coherent grain boundary, as confirmed by using STEM–energy-dispersion spectroscopy mapping. Our calculations indicate that the diffusion of Ni²⁺ induced by high-temperature activation plays a significant role in facilitating the phase separation. The relatively large chemically separated two-phase structure is expected to exhibit different performance characteristics compared with the previously reported nanosized two-phase structures, providing a new foundation for further improving high-energy-density LMR cathodes.

## Teaser

For the first time, a chemically separated two-phase structure has been discovered in an Li-rich cathode. This novel phenomenon, driven by temperature-dependent ion migration, significantly impacts its electrochemical properties.

## INTRODUCTION

Lithium-manganese-rich (LMR) oxides are emerging as highly promising candidates for next-generation cathodes, primarily due to the abundant availability of manganese resources and their impressive energy density. Compared with the conventional LiTMO_2_ (TM = transition metal) layered oxide cathode, the dual exploitation of both reversible cationic and anionic redox processes in LMR allows the extraction of more lithium ions [1–3]. Therefore, commercial batteries with LMR cathodes can reach up to 600 Wh/kg [4–8]. However, the enhanced capacity entails significant compromises on other vital electrochemical performances, including inadequate rate capability, irreversible capacity during initial charge cycles, intrinsic voltage hysteresis, accelerated capacity fade and rapid voltage decay [9,10]. These challenges, unfortunately, cannot be mitigated merely by applying modification strategies that have been successful for nickel-rich layered cathodes, owing to the fundamental differences in lattice and electronic structures between them [11,12]. This requires us to optimize the solid-state synthesis procedure of LMR oxides, such as the ratio of Li and TM, the precursor type and the annealing temperature, which significantly influence the structure of LMR oxides. In this sense, it is imperative to understand the microscopic structure of LMR oxides and establish the convincible structure–performance correlation in order to effectively improve the performances of LMR oxides.

However, the microscopic structure of LMR oxides remains a subject of debate. Thackeray *et al.* propose that the LMR cathode comprises two distinct phases that are intermixed at the nanoscale with Li_2_MnO_3_ and LiNi*_x_*Co*_y_*Mn_1__–_*_x_*_–_*_y_*O_2_ structures (two-phase model) [13], in which the coherent TM layer exhibits a sharp shift from a lithium-rich ordering to a lithium-deficient region, resulting in a disparity in symmetry [14–17]. Conversely, Lu *et al.* suggested another model in which these excess Li ions and TM ions coalesce at the atomic scale (solid-solution model) [18] and, in this scenario, lithium ions within the TM layer form a randomly distributed honeycomb superlattice [19–21]. Consequently, there are two kinds of viewpoints when interpreting the correlation between structure and cycling performance, based on these two microscopic structure models of LMR oxides, respectively. For instance, Liu *et al*. demonstrate that partial delithiation in one phase precipitates significant strain at the domain interface [15]—a phenomenon based on the two-phase model. Conversely, Wang *et al*. found that the presence of stacking faults plays a crucial role in elevating O 2p electron energy and thus enhances oxygen redox activation, as suggested by a solid-solution model [22]. To resolve the nanoscale structure of LMR oxides [22,23], transmission electron microscopy (TEM) methods have been given great attention to retrieve the atomic arrangement of the lattice in real space. However, distinguishing between the two models is challenging, as the projected nanosized two-phase model can exhibit lattice contrast that is identical to that of an overlapped solid-solution model in high-resolution scanning transmission electron microscopy (STEM) imaging, particularly when stacking faults are assumed [24]. Despite its importance, the microstructure of LMR oxides remains an unresolved scientific puzzle that has persisted for over two decades.

**Figure 1.**
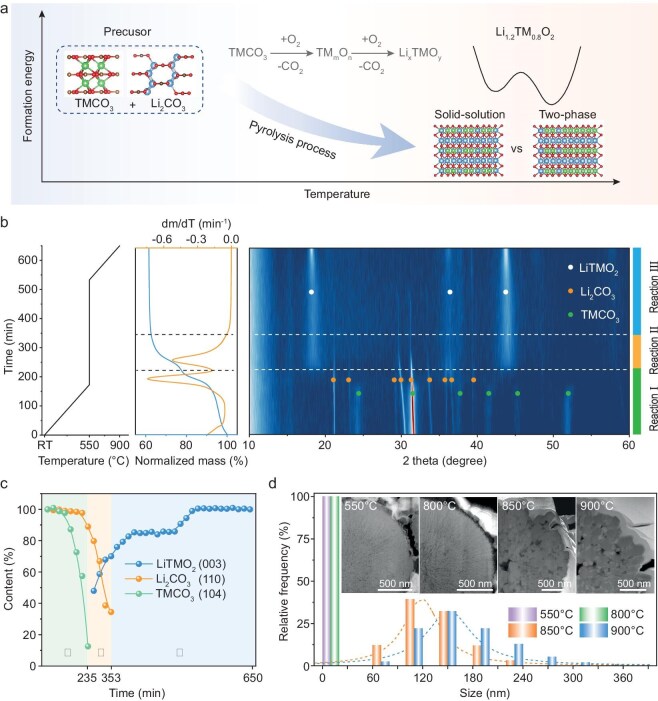
(a) Schematic representation of the phase-structure evolution during the LMR cathode material growth process. (b) TGA curves, including the temperature ramp profile and corresponding *in situ* XRD counterplots. (c) Rate of change in the integrated peak areas of XRD characteristics for LiTMO_2_, Li_2_CO_3_ and TMCO_3_. (d) Cross-sectional SEM images of LMR products at different temperatures, along with their corresponding particle-size distributions.

In this study, we explore the pyrolysis pathways and microscopic structures during the solid-state synthesis of Li_1.2_Ni_0.2_Mn_0.6_O_2_ cathodes. Through *in situ* X-ray diffraction (XRD), we identify the nucleation of solid-solution layered oxides alongside secondary phases from mixed Ni-/Mn-/Li-carbonate precursors at 550°C. As the temperature rises to 800°C, the nanoscale *C2/m* domains grow larger and the secondary phases vanish. When the temperature increases from 850°C to 900°C, analytical TEM results reveal a transition from a solid-solution structure (*C2/m*) to a chemically separated two-phase (CSTP) structure (*C2/m* and *R3̅m*), as illustrated in Fig. 1a. Furthermore, computational results and thermogravimetric analysis (TGA) have been performed to correlate the diffusion of ions upon annealing. This study not only contributes to resolving the longstanding debate surrounding the structure of LMR cathodes, but also provides critical insights for their precise design and optimization.

## RESULTS AND DISCUSSION

To investigate the structure evolution during the synthesis of LMR cathodes, we employed Ni/Mn metal carbonates along with lithium carbonate as precursors (see experimental section for detailed synthesis parameters). In order to probe the structural changes during synthesis, we utilized TGA and an *in situ* XRD method to monitor the reaction between TMCO_3_ and Li_2_CO_3_, as illustrated in Fig. 1b. Following the typical procedure, the sample was heated in air from room temperature to 550°C at a rate of 2°C/min, held at this temperature for 6 hours and subsequently heated to 900°C at the same rate. The TGA results revealed two distinct mass loss regions, reflected by two peaks in the derivative curve, enabling the reaction to be divided into three stages (Reactions I, II and III), as indicated by the dashed lines. During Reaction I (0 min to 235 min), TMCO_3_ is supposed to decompose primarily into TMO*_x_*. Meanwhile, the diffraction peaks of Li_2_CO_3_ show no significant intensity reduction, indicating that Li_2_CO_3_ is in its stable phase. Notably, the diffraction peaks of TMO*_x_* are weakly discernible, likely due to their very small grain size and poor crystallinity. Based on the evolution of diffraction peaks and mass loss data, an oxygen-involved decomposition mechanism of TMCO_3_ is proposed, as outlined by the following equation:


(1) \begin{eqnarray*}
0.8{\mathrm{TMC}}{{\mathrm{O}}}_3 + 0.14{{\mathrm{O}}}_2 = {\mathrm{T}}{{\mathrm{M}}}_{0.8}{{\mathrm{O}}}_{1.08} + 0.8{\mathrm{C}}{{\mathrm{O}}}_2.
\end{eqnarray*}

Following Equation (1), TMCO_3_ is transformed into TMO*_x_* with an average TM valence of 2.7. Notably, previous study [25] indicates that Ni ions at this temperature exhibit a valence of <2, suggesting that Mn ions are more readily oxidized than Ni ions and act as oxygen scavengers during the synthesis process. Upon a further increase in the temperature to 550°C, Reaction II occurs between Li_2_CO_3_ and TMO*_x_*. Although Li_2_CO_3_ decomposes at temperatures of >700°C [26], this reaction still proceeds due to the lower energy barrier for TMO*_x_* lithiation. The insertion of Li ions induces the formation of a layered structure, as evidenced by the abrupt appearance of its (003) diffraction peaks. However, the broad full width at half maximum (FWHM) of these peaks suggests a small grain size, indicative of significant crystal defects. The equation for Reaction II is therefore calculated as follows:


(2) \begin{eqnarray*}
{\mathrm{T}}{{\mathrm{M}}}_{0.8}{{\mathrm{O}}}_{1.08} &+& \ 0.456\,{\mathrm{L}}{{\mathrm{i}}}_2{\mathrm{C}}{{\mathrm{O}}}_3 + 0.03\,{{\mathrm{O}}}_2\\
&&= {\mathrm{L}}{{\mathrm{i}}}_{0.912}{\mathrm{T}}{{\mathrm{M}}}_{0.8}{{\mathrm{O}}}_{1.596} + 0.456\,{\mathrm{C}}{{\mathrm{O}}}_2.\\
\end{eqnarray*}

During Reaction II, the oxidation of transition metal (TM) ions is constrained, resulting in an average TM valence of 2.85. This limitation hinders the full transition from TMO*_x_* to the ideal layered structure. Upon a further temperature increase, the activation of oxygen gas facilitates the oxidation of TM ions, promoting the enhanced crystallization of the layered structure, as represented by the equation for Reaction III:


(3) \begin{eqnarray*}
{\mathrm{L}}{{\mathrm{i}}}_{0.912}{\mathrm{T}}{{\mathrm{M}}}_{0.8}{{\mathrm{O}}}_{1.596} &+& 0.144\,{\mathrm{L}}{{\mathrm{i}}}_2{\mathrm{C}}{{\mathrm{O}}}_3 + 0.13\,{{\mathrm{O}}}_2\\
&&= {\mathrm{L}}{{\mathrm{i}}}_{1.2}{\mathrm{T}}{{\mathrm{M}}}_{0.8}{{\mathrm{O}}}_2 + 0.144\,{\mathrm{C}}{{\mathrm{O}}}_2.\\
\end{eqnarray*}

Moreover, an elevated temperature promotes the grain growth, resulting in a reduced FWHM of the (003) diffraction peaks. However, the intensity of the (003) diffraction peak of the layered phase exhibits a sudden increment at >550°C (Fig. 1c). This abrupt reaction suggests that a higher temperature helps to overcome the energy barrier for the formation of the layered structure.

To investigate the structure evolution of the LMR cathode at higher temperatures, we held a mixture of Li_2_CO_3_ and TMCO_3_ at 550°C for 6 hours, followed by an additional annealing at 800, 850 and 900°C for 12 hours, respectively. The scanning electron microscopy (SEM) images in Fig. S1 reveal the grain-size changes at different temperatures. At 800°C, the small grains observed for the LMR oxides indicate insufficient conditions for crystal growth, whereas temperatures of 850°C and 900°C accelerate lattice fusion, resulting in a rapid increase in the grain size from nanoscale to the submicron scale for primary particles. The grain sizes are further quantified by the high-angle annular dark-field (HAADF) cross-sectional images in Fig. 1d. As shown in the XRD patterns of LMR samples synthesized at 850°C and 900°C (Fig. S2), the intensity ratio of (003)/(104) for LMR-900°C is 2.86, which is higher than that of LMR-850°C, suggesting reduced Li/TM-ion mixing. However, the separation of the Li_2_MnO_3_ and LiTMO_2_ structures also influences the degree of ion mixing. Ion mixing in the LMR system cannot be solely attributed to the valence of the Ni ions, as previously proposed for Ni-rich cathodes [27,28], and further atomic-scale investigations are essential to fully understand the element migration during annealing.

The nanosized LMR phase at 550°C accompanies secondary phases as well as defects. The HAADF images in Fig. S3 reveal nano-phases that predominantly consist of phases of rock salt, spinel, layered and Li_2_MnO_3_, distinguished by the degree of ion mixing in the TM/Li layers and the ordering within the TM layer. The phases share coherent interfaces, transiting from one ordering to another, and are readily interconverted by ion migration, indicating a competitive formation process. Despite phase separation, the elemental distribution and valence states remain uniform across the structures. Fig. S4 displays the distribution of Ni, Mn and O at different scales, highlighting the homogeneous mixing of elements from the nano- to microscale within the secondary particle. Furthermore, the electron energy-loss spectra (EELS) in Fig. S5 suggest a consistent oxidation state across the phases, with unchanged pre-peak intensity at the O-K edges and similar peak profiles for the Mn-L_2,3_ edges, respectively. These defected phases gradually transform into a single phase upon further heating to 800°C despite the trifle remaining spinel-like phase (Fig. S6). As shown in Fig. S7, the uniform distributions of Ni, Mn and O as well as the small grain size in LMR-800°C indicate that 800°C is insufficient for crystal growth. However, these nanocrystals undergo internal ion redistribution and rotational alignment, indicated by the HAADF images in Fig. S8, leading to the formation of an Li_2_MnO_3_-like structure with well-ordered crystal orientation. In addition, it is noted that the grain size and phase are similar from the surface to the bulk of the secondary particle, indicating a spatially homogeneous lithiation and oxidation process (Fig. S9). Compared with LMR-550°C, the oxidation degree of LMR-800°C is significantly higher, as evidenced by the enhanced pre-peak intensity of the O-K edge (Fig. S10). The increased oxygen ion incorporation not only facilitates the transition from defected LMR phases to the Li_2_MnO_3_-like structure, but also promotes crystal orientation alignment. Collectively, these findings underscore the critical role of oxidation in the structural transition from LMR-550°C to LMR-800°C, and the grain growth necessitates a higher temperature to prompt long-range ion migration.

**Figure 2.**
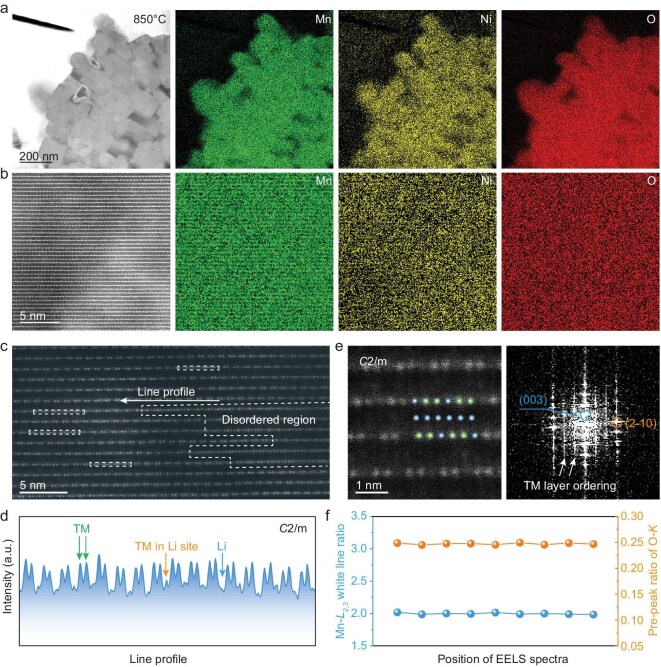
(a, b) STEM images of LMR-850°C with corresponding STEM–EDS elemental distributions of Ni, Mn and O at different magnifications. (c) High-resolution STEM image of LMR-850°C. (d) Line-profile analysis of the white-line intensities from the region shown in (c). (e) Enlarged view of atomic arrangements with the associated FFT pattern. (f) Quantitative analysis of the white-line ratio and oxygen pre-peak intensity derived from EELS spectra.

As shown in Fig. 2a, submicron grains are observed at 850°C, accompanied by a uniform Ni and Mn distribution at low magnification. Figure 2b presents the atomic-scaled STEM–energy-dispersion spectroscopy (EDS) mappings for Mn, Ni and O, showing their homogeneous distributions. The enlarged atomic arrangement in Fig. 2c shows the TM–TM–Li–TM–TM configuration as the ordered TM layer transits abruptly to a disordered state within a few nanometers. Even within the ordered regions, ion mixing is evident, as indicated by increased intensity at Li sites due to substantial TM-ion occupation (Fig. 2d). The disordered TM layers are attributed to either local TM/Li inhomogeneity or overlapping nanodomains with varying orientations. Nevertheless, the overall particle preserves a solid-solution phase with a *C2/m* space group, as confirmed in the fast Fourier transform (FFT) and selected electron diffraction patterns (Fig. 2e and Fig. S11). Moreover, the TM valence shows negligible variation across areas of slight elemental nonuniformity, as indicated by consistent oxygen pre-peak ratios and Mn white-line ratios (Fig. 2f and Fig. S12) [29].

**Figure 3.**
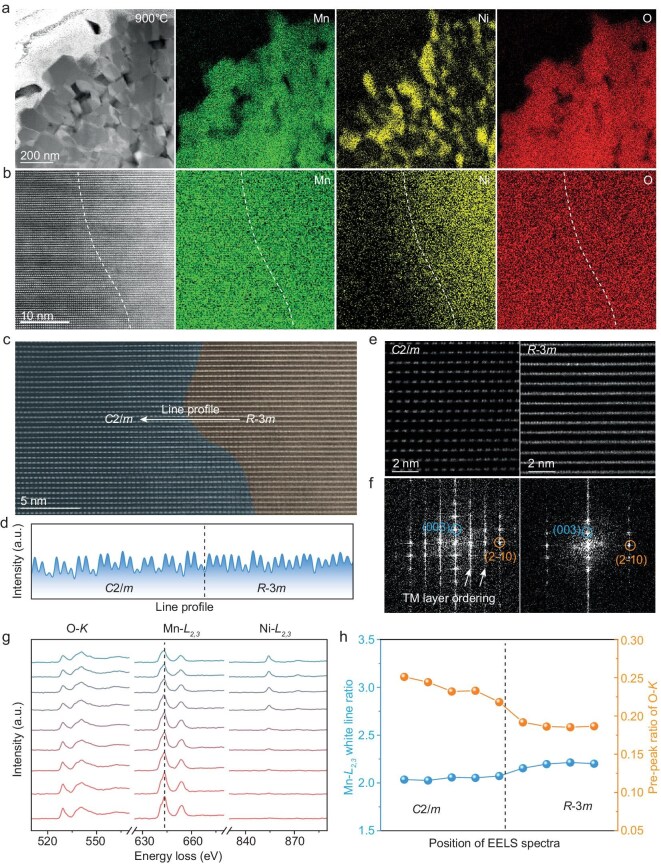
(a, b) STEM images of LMR-900°C with corresponding elemental distributions of Ni, Mn and O at varying magnifications. (c) High-resolution STEM image of LMR-900°C. (d) Line-profile analysis of the white-line intensities from the region shown in (c). (e) Enlarged HAADF–STEM images of atomic arrangements of *C2/m* and *R-3m* phases, respectively. (f) Corresponding FFT patterns of (e). (g) EELS spectra of O-K, Mn-L and Ni-L edges. (h) White-line ratio and oxygen pre-peak intensity, respectively, derived from EELS spectra (g).

A further increase in the temperature to 900°C intensifies long-range ion migration, resulting in pronounced Mn and Ni segregation, as shown by the STEM–EDS mappings in Fig. 3a. Figure 3b shows Ni-deficient and Ni-rich regions separated with white dished lines within one grain. The Ni-deficient regions appear darker in the HAADF images, reflecting a lower average atomic number due to higher Li-ion accumulation, while brighter Ni-rich regions result from Li-ion depletion caused by the ionic exchange between Ni and Li. Despite elemental nonuniformity, these regions maintain a coherent layered structure (Fig. 3c), with line profiles revealing a gradual transition from ordered TM layers to a disordered structure (Fig. 3d). The absence of Ni in the ordered regions indicates the formation of an Li_2_MnO_3_ phase with a space group *C2/m*, while Ni-rich regions correspond to an LiNi_0.5_Mn_0.5_O_2_ phase with a space group *R-3m*, as shown in the enlarged HAADF–STEM images of Fig. 3e. The LiNi_0.5_Mn_0.5_O_2_ and Li_2_MnO_3_ phases are confirmed by results from both the EDS quantification in Fig. S13 and the corresponding FFT patterns in Fig. 3f. Phase separation is also accompanied by changes in the reduced oxygen pre-peak intensity and Mn white-line ratio across the interfaces, as revealed by the EELS spectra in Fig. 3g. The white-line ratio changes from 2.2 for the LiNi_0.5_Mn_0.5_O_2_ phase to 2.0 for the Li_2_MnO_3_ phase, indicating lower Mn valence in the Ni-rich regions (Fig. 3h) [30]. While there are still some residual Ni ions in the C*2/m* phase due to the incomplete ion migration, we still refer to it as Li_2_MnO_3_ for convenience due to the negligible Ni content. Given the similarity in Mn white-line ratios between the Li_2_MnO_3_ phase and LMR-850°C, it is deduced that phase separation is affected by oxygen loss at high temperatures, facilitating the formation of the LiNi_0.5_Mn_0.5_O_2_ structure. A further increase in the sintering temperature to 1000°C does not alter the phase-separation characteristics of the LMR cathode, as shown in Fig. S14. This observation suggests that the formation of the two-phase structure remains thermally stable even at higher temperatures. These findings demonstrate a distinct structural transition of the LMR cathode materials from the solid-solution phase to the CSTP structure, involving ion migration and altered oxidation behavior.

We then compared the electrochemical performance of the samples sintered at 800°C, 850°C and 900°C, respectively. Fig. S15 shows the initial charge–discharge profiles for the LMR cathodes annealed at 800°C, 850°C and 900°C, respectively. Compared with the LMR-900°C cathode with a mere 190-mAh/g discharge capacity, the LMR-800°C and 850°C cathodes manifest a larger capacity surpassing 230 mAh/g. This difference is attributed to their phase diversity: a solid-solution structure favors the electrochemical activity of the LMR cathode, which is however restricted in the CSTP structure. Prior studies have shown that the Li_2_MnO_3_ cathode delivers a minor discharge capacity (<100 mAh/g) [31], indicating that the majority of the discharge capacity in the LMR-900°C cathode originates from the LiNi_0.5_Mn_0.5_O_2_ component. In contrast, the solid-solution phase disrupts the long-range cationic ordering in Li_2_MnO_3_, stabilizing the reversible oxygen redox and thereby boosting capacity [32,33]. Furthermore, the LMR-900°C cathode demonstrates accelerated voltage decay, as shown in Fig. S16, indicative of a severely distorted Li_2_MnO_3_ lattice during cycling owing to its worse redox stability.

**Figure 4.**
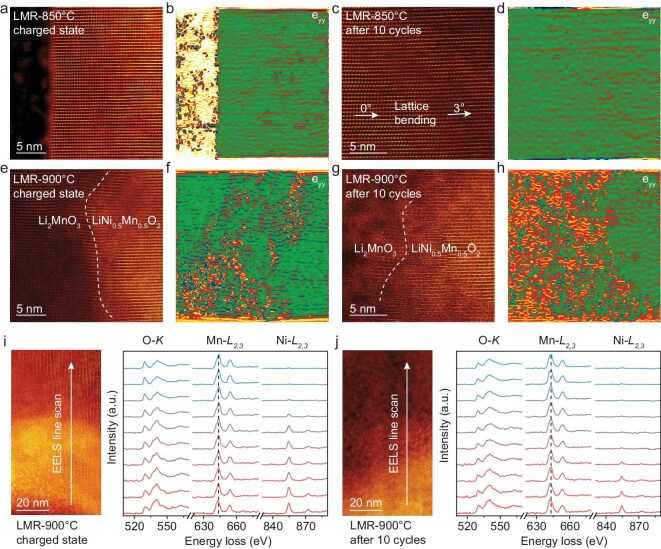
High-resolution STEM image of (a) LMR-850°C at charged state, (c) LMR-850°C at discharged state after 10 cycles, (e) LMR-900°C at charged state and (g) LMR-900°C at discharged state after 10 cycles. Corresponding GPA analyses are shown in (b, d, f, h), respectively. (i, j) EELS spectra of O-K, Mn-L and Ni-L edges for LMR-900°C at (i) charged state and (j) discharged state.

To elucidate the correlation between the structural evolution and electrochemical properties of these LMR cathodes, we investigated the structural changes of the LMR-850°C and LMR-900°C cathodes under different electrochemical conditions. As shown in Fig. S17, the LMR-850°C cathode exhibits a well-defined solid-solution structure, with only minor ion mixing observed at the surface. Upon charging the cathode to 4.7 V, we detected a slight lattice distortion due to delithiation (Fig. 4a), which is further supported by the geometric phase analysis (GPA) (Fig. 4b). On the other hand, the EELS results of the O-K edge from the charged state depicted in Fig. S18 shows a significant increase in the pre-peak. After 10 cycles, various lattice defects emerged, including lattice bending, antiphase boundaries and spinel-like structures, as illustrated in Fig. 4c and Fig. S19, respectively. Despite these structure distortions, the majority of the particle maintained a *C2/m* structure with mitigated strain, as shown by GPA in Fig. 4d, which minimizes voltage decay during cycling.

In contrast, the LMR-900°C cathode with separated phases experienced more severe lattice distortion during cycling. Figs S20 and S21 depict the structural changes of the LMR-900°C cathode from its pristine state to a charged state at 4.7 V. The well-crystallized regions of LiNi_0.5_Mn_0.5_O_2_ and Li_2_MnO_3_ became defected as a result of delithiation. Specifically, the LiNi_0.5_Mn_0.5_O_2_ structure exhibited bended lattice, while the Li_2_MnO_3_ structure developed numerous defects, including antiphase boundaries, spinel-like phases and even dislocations. Additionally, the mismatch between the delithiated phases generated significant strain at the interface, leading to complex lattice distortions in the boundary area, as revealed in Fig. 4e and f [15]. After 10 cycles, the LiNi_0.5_Mn_0.5_O_2_ region retained its layered structure, as shown in Fig. S22. However, the pronounced oxygen redox process caused severe mass loss within the Li_2_MnO_3_ structure, resulting in voids and poorly crystallized structure [34]. Consequently, the strain at the interface between the two phases became more pronounced, as shown in Fig. 4g and h. In addition to lattice variations, the valence of the TM was also reduced following electrochemical cycling. By comparing the EELS results for the LMR-900°C cathode before cycling, at charged state and after cycling, we observed significant oxygen loss in the Li_2_MnO_3_ region, as indicated by the reduced intensity of its O-K edge pre-peak after 10 cycles (Fig. 4i and j). These findings confirm that the LMR-900°C cathode undergo severe structural degradation, attributed to both the intrinsic electrochemical instability of Li_2_MnO_3_ and the substantial strain between the phases resulting from lattice mismatch during cycling [35]. In contrast, the uniform dispersion of Ni ions in the solid-solution structure effectively inhibits structural changes, thereby promoting the cycling stability of the LMR cathode.

**Figure 5.**
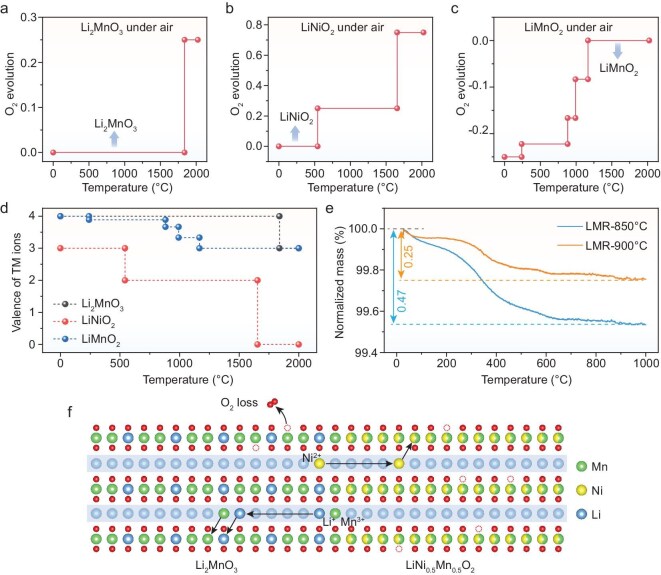
(a–c) Thermodynamic calculations of oxygen evolution during the heating of Li_2_MnO_3_, LiNiO_2_ and LiMnO_2_ in an air atmosphere. (d) Valence change of Li_2_MnO_3_, LiNiO_2_ and LiMnO_2_ calculated according to the thermodynamic stable phases. (e) Temperature-dependent mass change profiles for LMR-850°C and LMR-900°C under an air atmosphere. (f) Schematic representation of the phase transitions occurring in the LMR cathode materials during its thermal evolution.

To elucidate the driving force of the temperature-dependent structural evolution of the LMR materials, first-principles calculations were performed to evaluate the thermodynamic behavior of the system as a function of temperature, obtaining stable phases of relevant compositions in an air atmosphere across varying temperatures (Fig. 5a–c and Fig. S23). Calculation results indicate that the favored oxidation states of TMs in oxides depend on temperature. Specifically, increasing temperatures generally lower the valence states of TMs, leading to the release of O_2_ gas (Fig. 5d). The Li_2_MnO_3_ phase was found to remain thermodynamically stable up to 1800°C, which explains the persistence of TM-layer ordering even at a relatively low temperature of 550°C. In contrast, the LiNiO_2_ and LiMnO_2_ phases exhibit instability at >550°C. Between 800°C and 900°C, Mn^3+^ is oxidized to higher valence states, whereas Ni³⁺ tends to be reduced to Ni²⁺, favoring the formation of LiNi_0.5_Mn_0.5_O_2_ after phase separation. From a kinetics perspective, the reduction of Ni³⁺ to Ni²⁺ lowers the migration-barrier nickel cations by decreasing the electrostatic energy. This reduction in the migration barrier likely contributes to phase separation as the temperature rises, consistently with experimental observations showing that such phase separation occurs at >850°C. The structural transformation is therefore driven by both thermodynamic and kinetic factors. Additionally, this valence-state-dependent cation migration sheds light on the general stability challenges associated with high-Ni cathode materials, as the reduction of the Ni valence state increases the likelihood of nickel-ion diffusion, further impacting material stability.

Ion migration requires not only elevated temperatures, but also lattice defects, such as vacancies, as well as a suitable ion valence to minimize the diffusion barriers. To probe ion-diffusion-induced phase separation, TGA was conducted for LMR-850°C and LMR-900°C in pure O_2_ (Fig. 5e). Both samples exhibited gradual mass loss due to oxygen release with increasing temperature. Given that the two materials would reach similar oxygen-vacancy concentrations in their final states, the more pronounced O_2_ release from LMR-850°C indicates a lower initial concentration of oxygen vacancies. It is thereby concluded that, for LMR-900°C, oxygen is more severely expelled from the structure, accompanied by TM-ion reduction to maintain charge neutrality. This reduction is evident in the EELS spectra of Fig. S24, in which a shift in the Ni-L_2,3_ peaks to lower energy is observed with increasing temperature. In addition, the X-ray photoelectron spectroscopy (XPS) results in Fig. S25 also show that, with increasing temperature, the content of oxygen vacancies would increase due to lattice oxygen release.

Based on the above results, we propose a phase-transition mechanism for the LMR cathode based on different ionic diffusivity at high temperature, as shown in the model of Fig. 5f. At 550°C, a nanosized LMR phase forms, and Ni and Mn ions remain intermixed due to restricted ion migration, resulting in high spatial inhomogeneity with varying degrees of ordering. At 850°C, short-range ion migration becomes feasible, enabling the transformation of these nanodomains into a relatively stable state of an Li_2_MnO_3_-like solid-solution structure with TM-layer ordering. Further elevation of the temperature to 900°C triggers a slight oxygen release from the lattice, leading to higher oxygen-vacancy concentrations and reduced Ni and Mn ions. The Ni and Mn ions with reduced valence have fewer coulombic interactions with surrounding ions, which facilitates their migration and induces the counter-migration of Li, Mn and Ni ions for charge compensation. Enhanced oxygen-vacancy formation further promotes the diffusion of cationic ions, ultimately leading to an energetically favorable CSTP structure. We believe that the oxygen release plays a critical role to affect the valence and thus the diffusivity of Ni ions during sintering.

## CONCLUSIONS

In summary, our study provides critical insights into the structural evolution of Li_1.2_Ni_0.2_Mn_0.6_O_2_ cathode materials during their synthesis, resolving a longstanding debate regarding their intrinsic structural characteristics. We found that the transformation begins at 550°C, when TM carbonates undergo a transformation into nanosized oxide phases, including rock-salt, spinel-like, layered and Li_2_MnO_3_-like phases. Between 550°C and 800°C, these nanosized phases progressively transition into a solid-solution structure (*C2/m*) characterized by homogeneous Ni- and Mn-ion distribution across particle and nanoscale levels. At 850°C, the temperature suffices to promote the growth of the solid-solution phase. Upon further heating to 900°C, we observed a CSTP structure emerging from the solid-solution phase. This CSTP structure comprising coherent Li_2_MnO_3_ and LiTMO_2_ phases is different from the nanoscale two-phase mixture reported in the literature [14]. Our calculations and TGA results indicate that an increase in the temperature to 900°C induces a reduction in the Ni valence via the formation of additional oxygen vacancies, facilitating Ni-, Mn- and Li-ion convection and ultimately resulting in phase separation into Li_2_MnO_3_ and LiNi_0.5_Mn_0.5_O_2_. The TM cations readjust their preferred oxidation states at elevated temperatures, altering the ground state and reducing the energy barriers for cation migration. This dynamic behavior results in phase transitions driven by both thermodynamic and kinetic factors. Our findings underscore the pivotal role of oxygen vacancies in enabling ion migration and offer novel insights for modulating phase composition and ordering in LMR cathode materials to improve electrochemical performance. As the phase composition of the material inherently determines its performance, targeted phase engineering of the CSTP structure may help to address the current challenges associated with LMR cathodes.

## MATERIALS AND METHODS

### Fabrication of LMR cathodes

The precursor nickel-manganese carbonate (Ni_0.25_Mn_0.75_CO_3_) was commercially sourced. For the synthesis of 0.01 mol of the LMR cathode material, 0.008 mol of the precursor was mixed with 0.006 mol of Li_2_CO_3_ (with a 5% excess) and ground for 20–30 min until a homogeneous mixture was obtained. The mixture was then subjected to calcination in a muffle furnace. The heating protocol involved ramping from room temperature to 550°C at a rate of 5°C min^–1^, followed by a dwell time of 360 min. Subsequently, the temperature was increased to 800°C, 850°C and 900°C at the same heating rate, with a holding time of 840 min at each target temperature, before natural cooling and sample collection.

### Materials characterization

XRD patterns and *in situ* XRD profiles were collected by using Rigaku SmartLab with Cu Kα radiation. SEM (ZEISS Gemini 500) was used to observe the morphology. Focused ion beam (FEI Helios 600i) was employed to prepare the thin-sample section for the STEM test. STEM–HAADF images and EDS mappings were obtained on an aberration-corrected JEOL NEOARM electron microscope operated at 200 kV. The EELS data were collected in a dual-EELS mode to obtain both zero-loss spectra and core-loss spectra by using a Gatan Imaging Filter (GIF) Quantum Model 1077 spectrometer. The TGA was obtained through a NETZSCH STA 449C. An XPS (PHI 5000) experiment was conducted to analyse the oxygen vacancies for different cathodes. The inductively Coupled Plasma-Optical Emission Spectroscopy (Agilent ICP-OES 725 ES) was applied to measure the practical ratio of Mn and Ni in the cathodes.

### Computational method

To evaluate the thermal stability of the Li–Mn(Ni)–O compounds, we constructed a series of grand potential phase diagrams at different oxygen chemical potentials (${\mu }_{{O}_2}$), from which the corresponding oxygen uptake or release critical values could be determined [36,37]. By assuming that the reaction entropy is only dominated by the gas phase, the effect of temperature and partial pressure can by captured by translating ${\mu }_{{O}_2}$ as follows [38,39]:


\begin{eqnarray*}
{{\mu }_{{O}_2}\!\left( {T,{p}_{{O}_2}} \right)}&=& { {\mu }_{{O}_2}\!\left( {T,{p}_0} \right) + {k}_BTln\frac{{{p}_{{O}_2}}}{{{p}_0}}}\\ && \approx {{E}_{{O}_2} + {k}_BT - TS_{{O}_2}^{T,{p}_0} + {k}_BTln\frac{{{p}_{{O}_2}}}{{{p}_0}},}
\end{eqnarray*}

where ${p}_{{O}_2}$ is the oxygen partial pressure, ${p}_0$ is the reference pressure, $S_{{O}_2}^{T,{p}_0}$ is the oxygen entropy, ${E}_{{O}_2}$ is the oxygen internal energy and ${k}_B$ is Boltzmann's constant. In this study, we set ${p}_{{O}_2}$ and ${p}_0$ to be 0.21 atm and 1 bar, respectively. ${E}_{{O}_2}$ was determined by extracting data from the Atomly.net database [40–42] and the experimental entropy data, $S_{{O}_2}^{T,{p}_0}$, were obtained from the NIST-JANAF thermochemical table [43].

## Supplementary Material

nwaf202_Supplemental_File
