# Supplementary material for: Revealing the chemical separated two-phase structure in lithium-manganese-rich cathode
Source: Natl Sci Rev. 2025 May 21;12(7):nwaf202. doi: 10.1093/nsr/nwaf202 (PMC12202208; doi:10.1093/nsr/nwaf202)
Supplement: nwaf202_Supplemental_File [file nwaf202_supplemental_file.pdf]

Supplementary Data

Revealing the chemical separated two-phase structure in lithium-manganese-rich cathode

Jiayi Wang[1,†], Xincheng Lei[2,3,†,*], Hao Meng[1], Pengxiang Ji[2,3], Tenglong Lu[2], Weijun Liang[4], Xiaozhi Liu[2], Sheng Meng[2,3,4], Lin Gu[5], Miao Liu[2,4,6,*], Xin Wang[1,*] and Dong Su[2,3,*]

[1]Institute of Carbon Neutrality, Zhejiang Wanli University, Ningbo, 315100 China;
[2]Beijing National Laboratory for Condensed Matter Physics, Institute of Physics, Chinese Academy of Sciences, Beijing, 100190 China;
[3]School of Physical Sciences, University of Chinese Academy of Sciences, Beijing, 100049 China;
[4]Songshan Lake Materials Laboratory, Dongguan, Guangdong 523808, China;
[5]Beijing National Center for Electron Microscopy and Laboratory of Advanced Materials, Department of Materials Science and Engineering, Tsinghua University, Beijing, 100084 China;
[6]Center of Materials Science and Optoelectronics Engineering, University of Chinese Academy of Sciences, Beijing 100049, China

***Corresponding authors.** E-mails: lxc@iphy.ac.cn; mliu@iphy.ac.cn; wangx@zwu.edu.cn; dongsu@iphy.ac.cn
†Equally contributed to this work.

Table of Contents

Figure. S1 SEM images for LMR particles annealed under different temperatures.	3
Figure. S2 XRD images for LMR particles annealed under different temperatures.	4
Figure. S3 HAADF images of LMR-550°C.	5
Figure. S4 EDS-mappings of LMR-550 ^{o}C.	6
Figure. S5 EELS of LMR-550 ^{o}C.	7
Figure. S6 HAADF images of LMR-800°C showing structure transition.	8
Figure. S7 EDS-mappings of LMR-800 ^{o}C.	9
Figure. S8 HAADF images of LMR-800 ^{o}C.	10
Figure. S9 Surface and bulk structure of LMR-800 ^{o}C.	11
Figure. S10 EELS of LMR-800 ^{o}C.	12
Figure. S11 Selected area electron diffraction of LMR-850 ^{o}C.	13
Figure. S12 EELS of LMR-850 ^{o}C.	14
Figure. S13 EDS quantification.	15
Figure. S14 Structure of LMR-1000 ^{o}C.	16
Figure. S15 Initial charge-discharge profiles for LMR cathodes.	17
Figure. S16 Comparison of voltage decay of LMR cathodes.	18
Figure. S17 HAADF and EELS for LMR-850 ^{o}C before cycling.	19
Figure. S18 EELS for LMR-850 ^{o}C at charged state.	20
Figure. S19 Structure of LMR-850 ^{o}C at discharged state after 10 cycles.	21
Figure. S20 HAADF and EELS for LMR-900 ^{o}C before cycling.	22
Figure. S21 Structure of LMR-900 ^{o}C at charged state.	23
Figure. S22 Structure of LMR-900 ^{o}C at discharged state after 10 cycles.	24
Figure. S23 Calculated stable phases under different temperatures.	25
Figure. S24 Comparison of Ni-$L_{2,3}$ edges for LMR-850 ^{o}C and LMR-900 ^{o}C.	26
Figure. S25 XPS results for LMR cathodes.	27

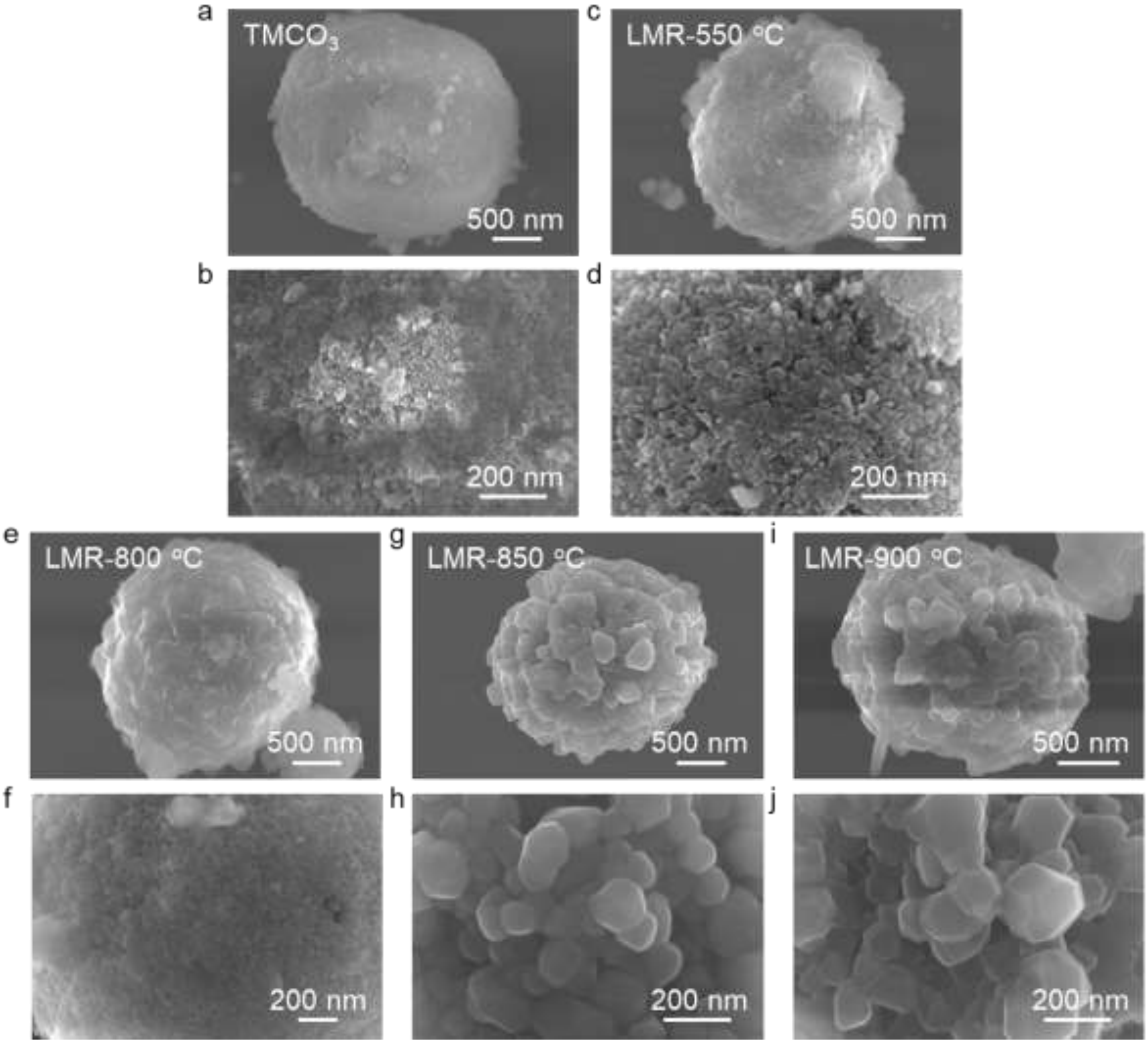

Figure S1. The SEM images of $TMCO_3$ and LMR cathode materials at different calcination temperature.

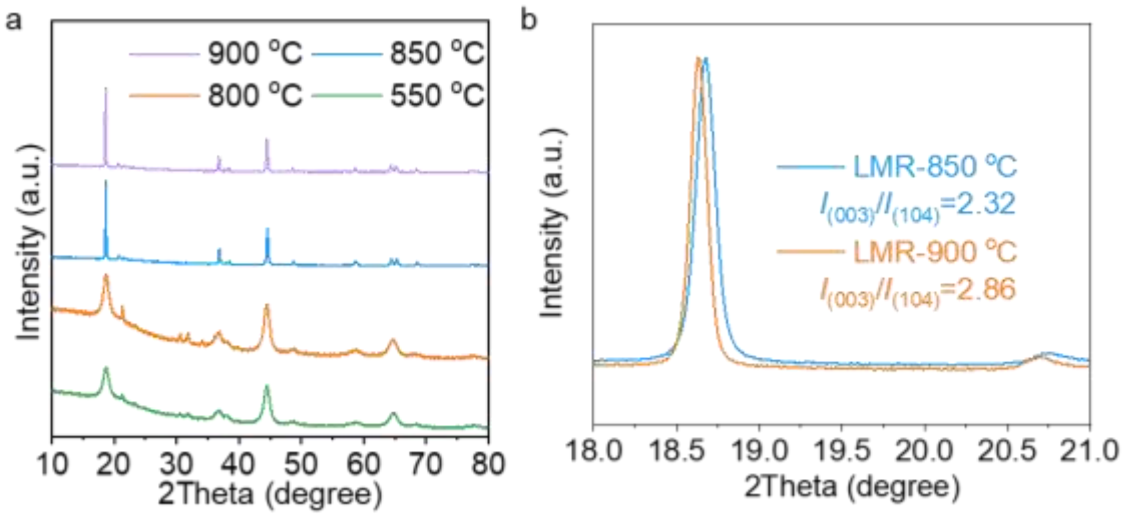

Figure S2. (a) The XRD patterns of LMR cathode materials at different calcination temperature and (b) the enlarged XRD patterns of LMR-850 ^{o}C and LMR-900 ^{o}C.

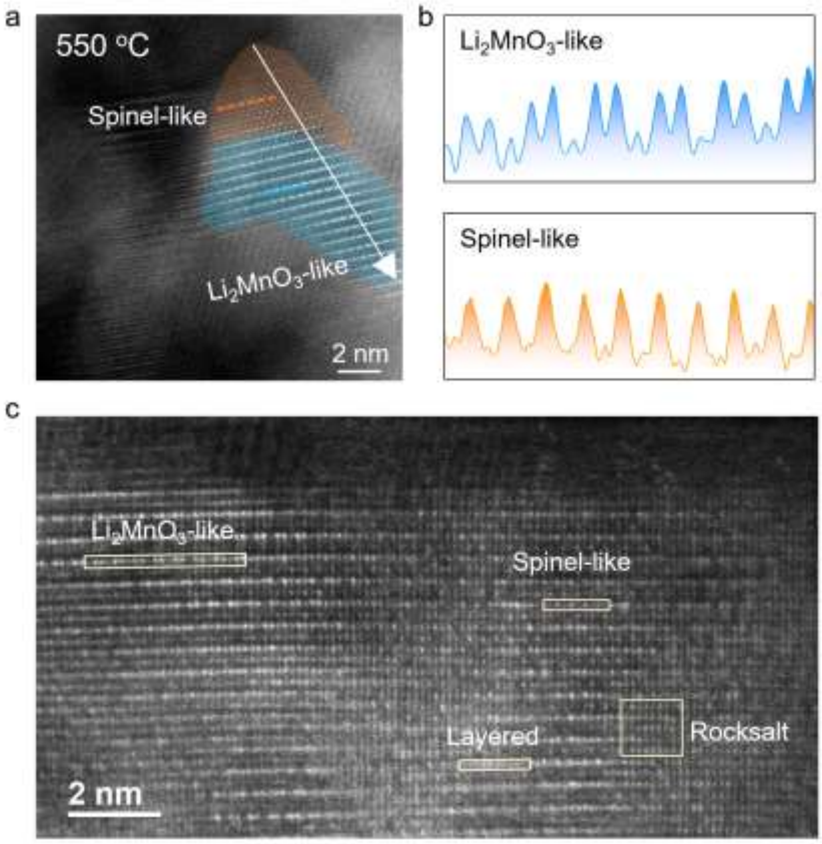

Figure S3. (a) HAADF image of LMR-550 ^{o}C showing the phase transition from spinel-like to Li_2MnO_3-like transition. (b) Line profile analysis from the region shown in (b). (c) HAADF image of LMR-550 ^{o}C showing the varied phases marked in yellow squares.

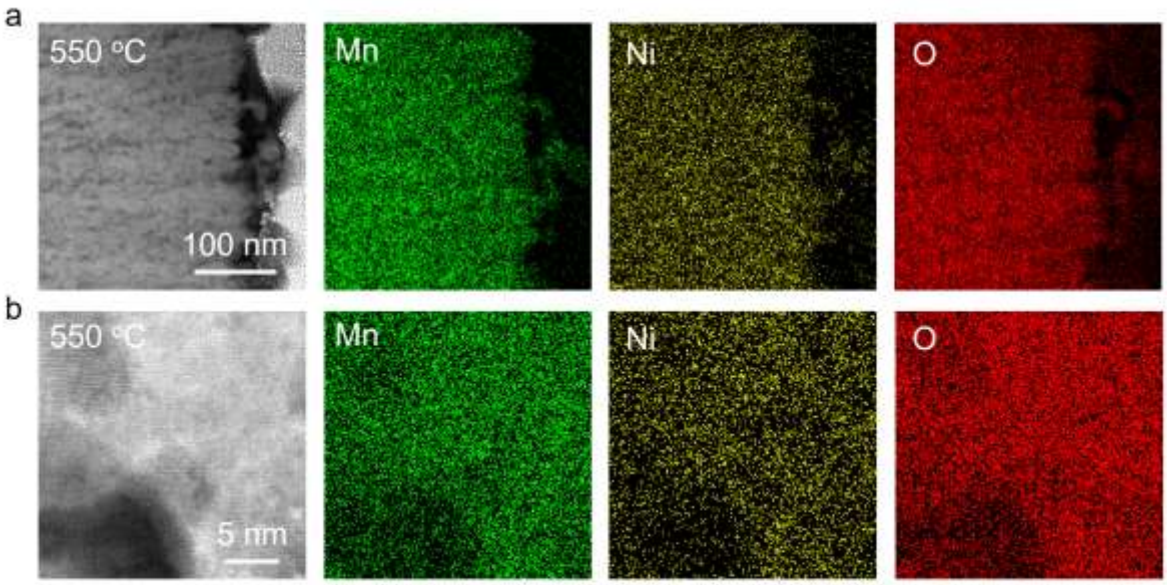

Figure S4. (a, b) STEM images of LMR-550 ^{o}C with corresponding elemental distributions of Ni, Mn, and O at varying magnifications.

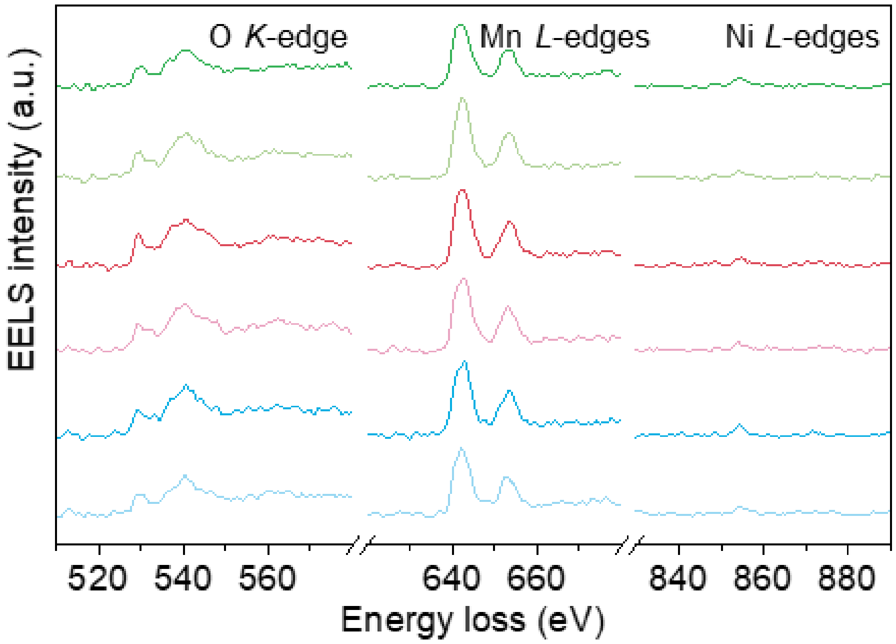

Figure S5. The EELS spectra of LMR-550 °C along the arrowed line in Figure S3.

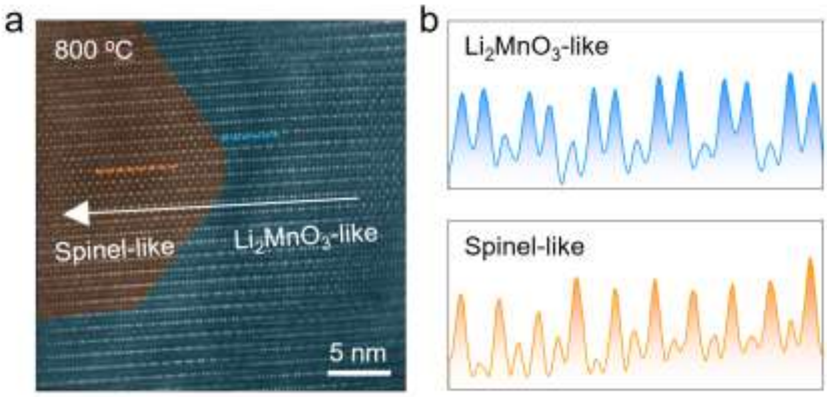

Figure S6. (a) High-resolution STEM image of LMR-800 °C. (b) Line profile analysis from the region shown in (b).

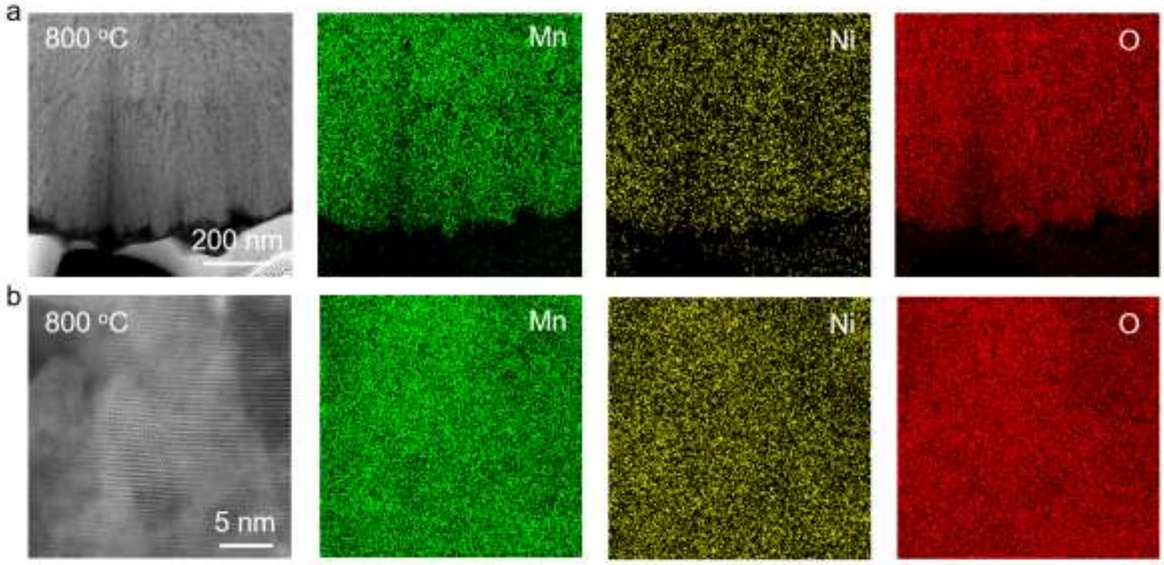

Figure S7. (a, b) STEM images of LMR-800 °C with corresponding elemental distributions of Ni, Mn, and O at varying magnifications.

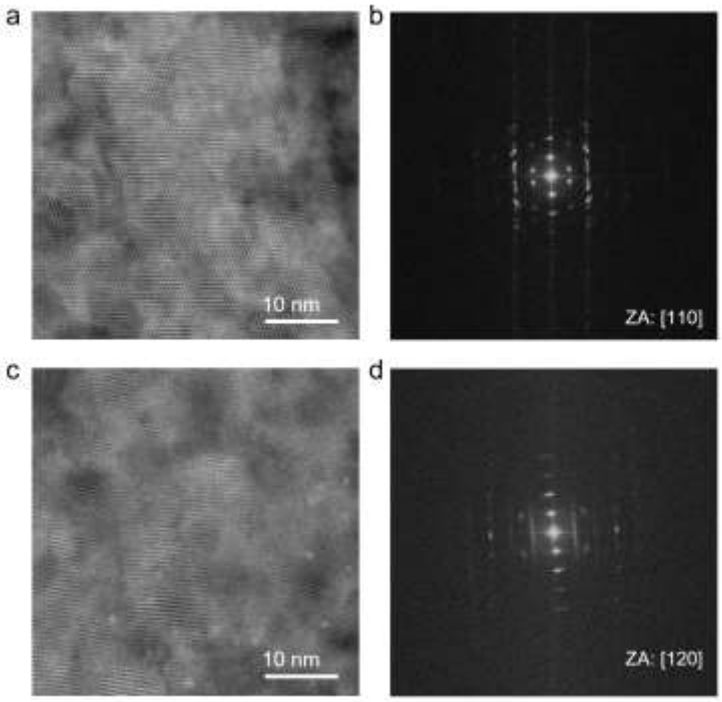

Figure S8. (a) HAADF image of LMR-800 ^{o}C in [110] zone axis and (b) corresponding FFT pattern. (c) HAADF image of LMR-800 ^{o}C in [120] zone axis and (d) corresponding FFT pattern.

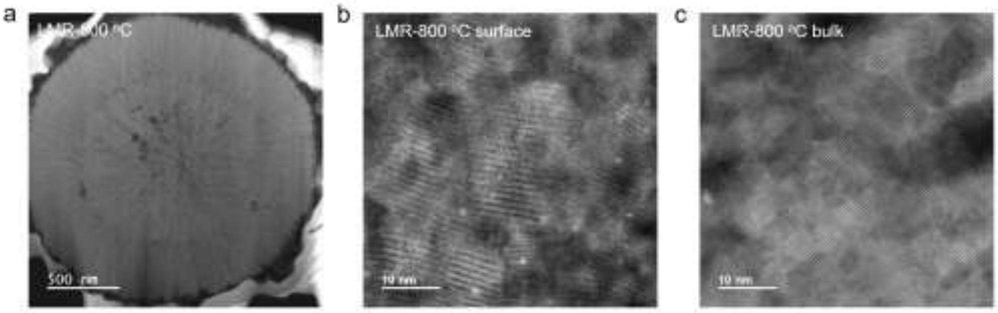

Figure S9. (a) HAADF image for LMR-800 ^{o}C cathode under low magnification. (b) The HAADF image for LMR-800 ^{o}C cathode near surface. (b) The HAADF image for LMR-800 ^{o}C cathode inside bulk.

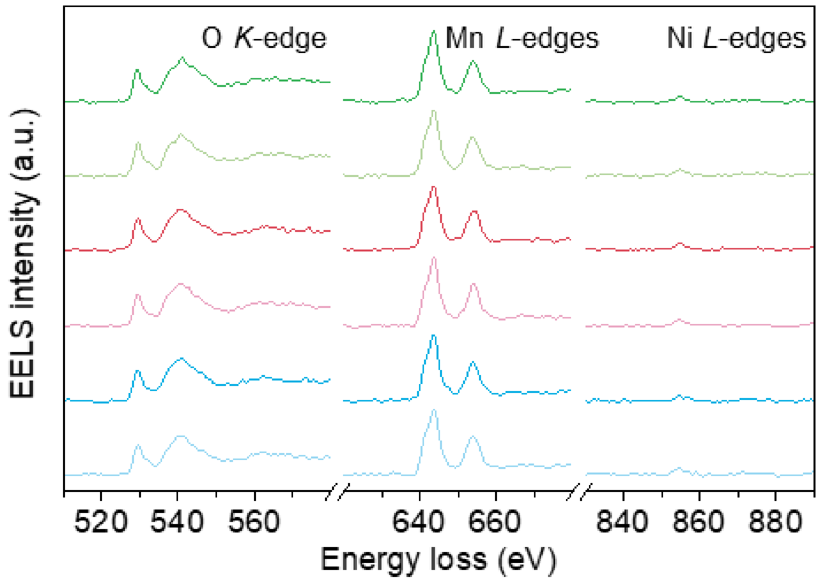

Figure S10. The EELS spectra of LMR-800 ^{o}C along the arrow line in Figure S6.

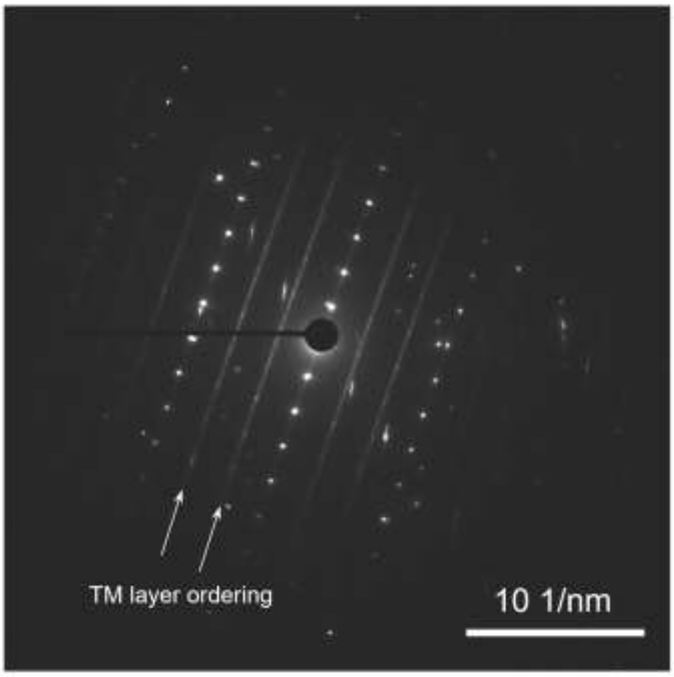

Figure S11. The selected electron diffraction patterns of LMR-850 ^{o}C.

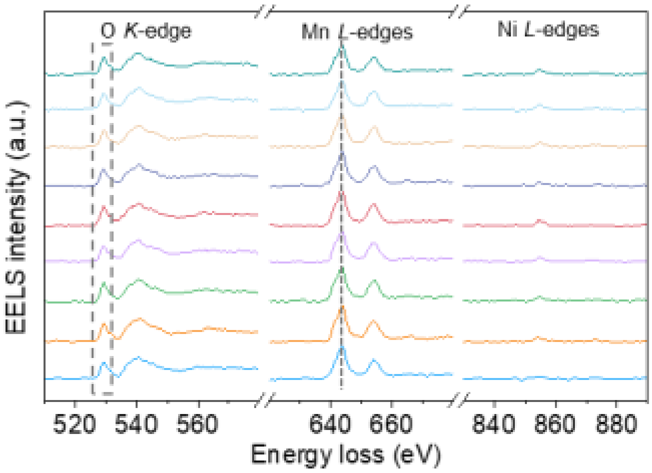

Figure S12. The EELS spectra of LMR-850 °C.

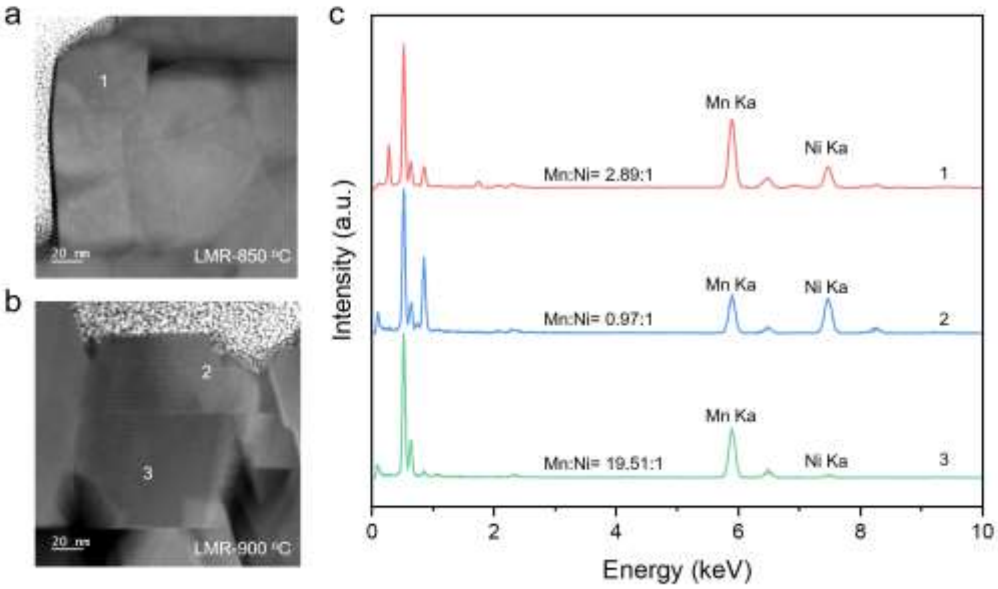

d

	EDS quantification		Normalized ratio	
	Mn	Ni	Mn	Ni
$Li_{1.2}Ni_{0.2}Mn_{0.6}O_2$	2.89	1	3	1
$LiNi_{0.5}Mn_{0.5}O_2$	0.97	1	1.01	1
Li_2MnO_3	19.51	1	20.25	1

Figure S13. Compositional quantification of LMR sample at different region. (a) and (b) are the HAADF images for LMR-850 ℃ and LMR-900 ℃, respectively, where the spectra are extracted in the positions marked with 1, 2 or 3. (c) The EDS spectra from different region. (d) Comparison of normalized Mn/Ni ratio from ICP between from EDS, in which the Mn/Ni ratio in LMR-850 ℃ cathode is set at 3:1 according to ICP results.

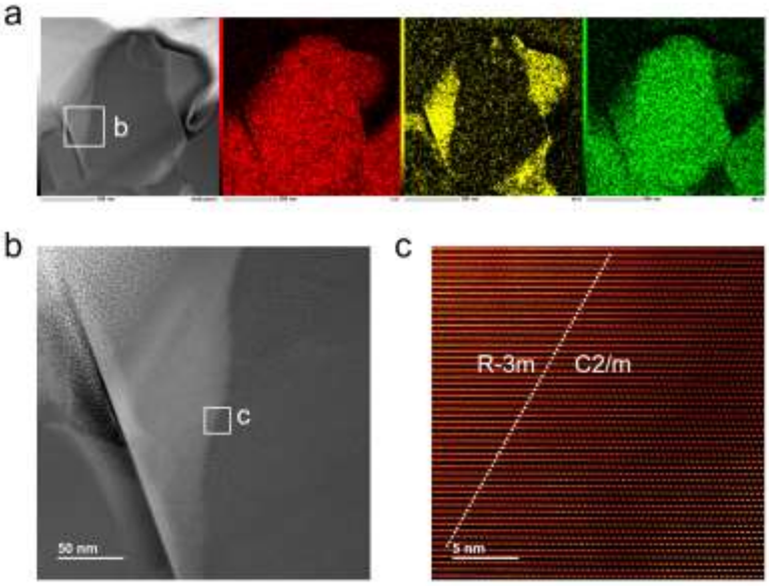

Figure S14. The structure of LMR cathode synthesized under 1000°C. (a) HAADF-STEM and STEM-EDS mappings showing the chemical segregation of Ni element. (b) HAADF image under low magnification. (c) High resolution HAADF image from the marked region in (b) showing the phase boundary between R-3m structure and C2/m structure.

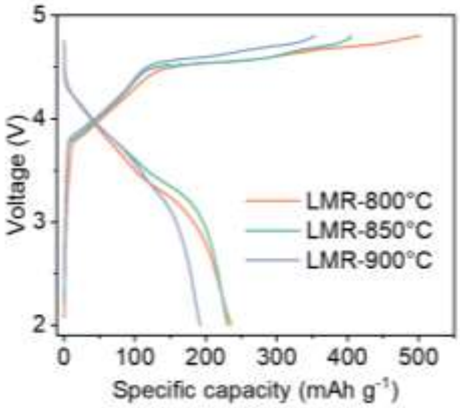

Figure S15. Initial charge-discharge curves for LMR cathodes synthesized under different temperatures.

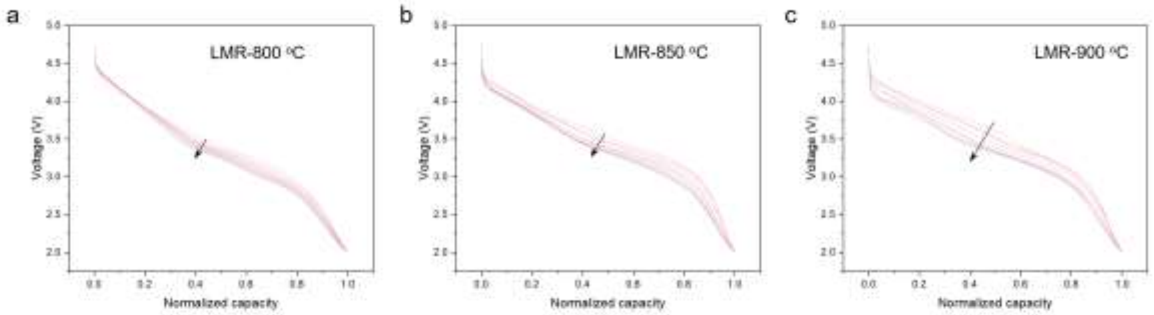

Figure S16. The Voltage decay for LMR cathodes synthesized under different temperatures.

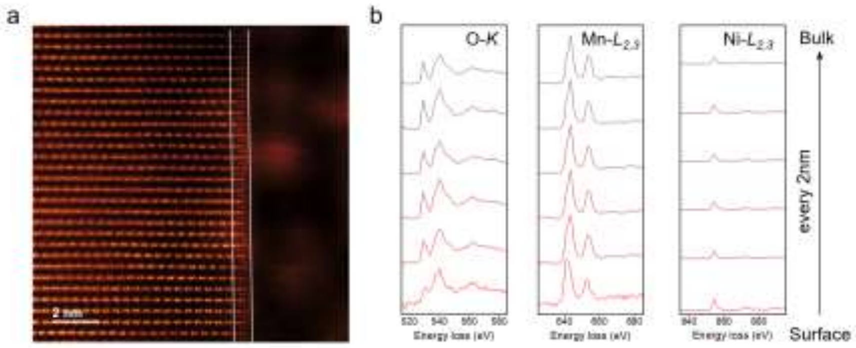

Figure S17. (a) HAADF-STEM image for LMR-850 ℃ cathode showing the structure of surface region. The marked subsurface region has a slight ion mixing. (b) STEM-EELS spectra of O-K, Mn-$L_{2,3}$ and Ni-$L_{2,3}$ peaks from surface to bulk.

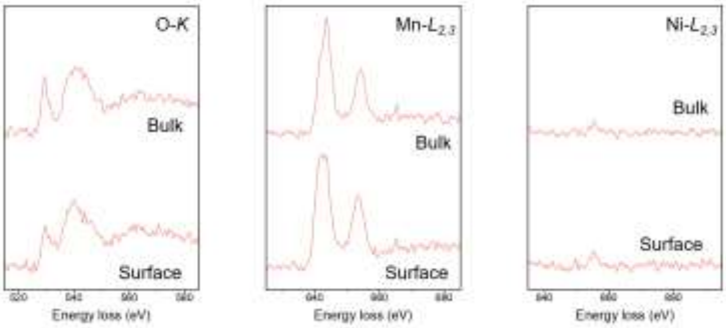

Figure S18. STEM-EELS spectra of O-K, Mn-$L_{2,3}$ and Ni-$L_{2,3}$ peaks taken from the surface and the bulk for LMR-850 ℃ cathode at charged state during initial cycling

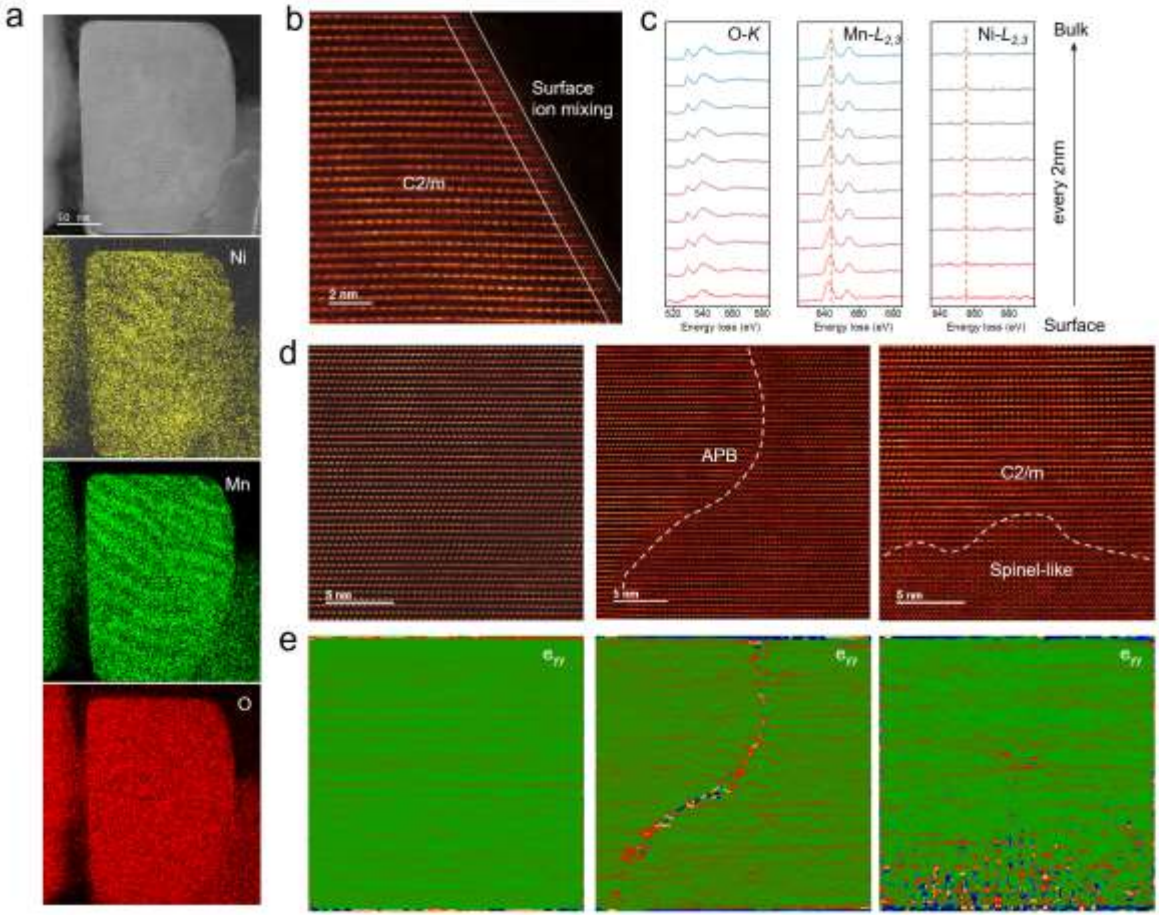

Figure S19. (a) STEM-EDS mappings for LMR-850 ℃ cathode at discharged state after 10 cycles showing the homogeneous distribution of Mn, Ni and O elements. (b) High resolution HAADF-STEM image showing the surface structure after cycling. (c) STEM-EELS line-scan spectra of O-K, Mn-$L_{2,3}$ and Ni-$L_{2,3}$ peaks from surface to bulk, respectively, with a step of 2nm. (d) High resolution HAADF-STEM images and (e) corresponding GPA analyses for the cycling-induced structure deformation after cycling.

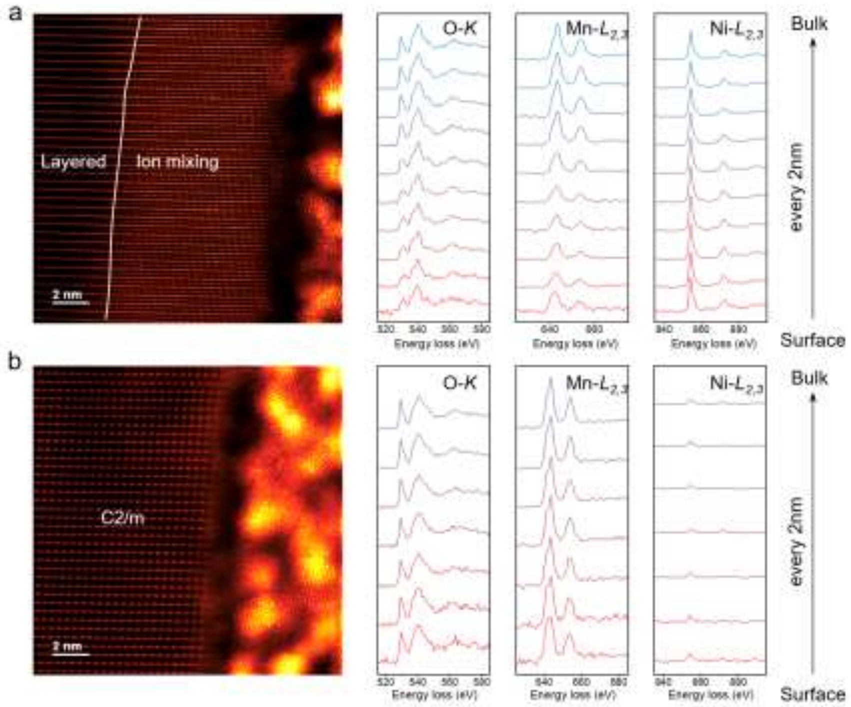

Figure S20. High resolution HAADF-STEM images showing the surface structure (left) and STEM-EELS spectra of O-K, Mn-$L_{2,3}$ and Ni-$L_{2,3}$ peaks from surface to bulk respectively, with a step of 2nm (right) of LMR-900 ℃ cathode. (a) $LiNi_{0.5}Mn_{0.5}O_2$ region. (b) Li_2MnO_3 region.

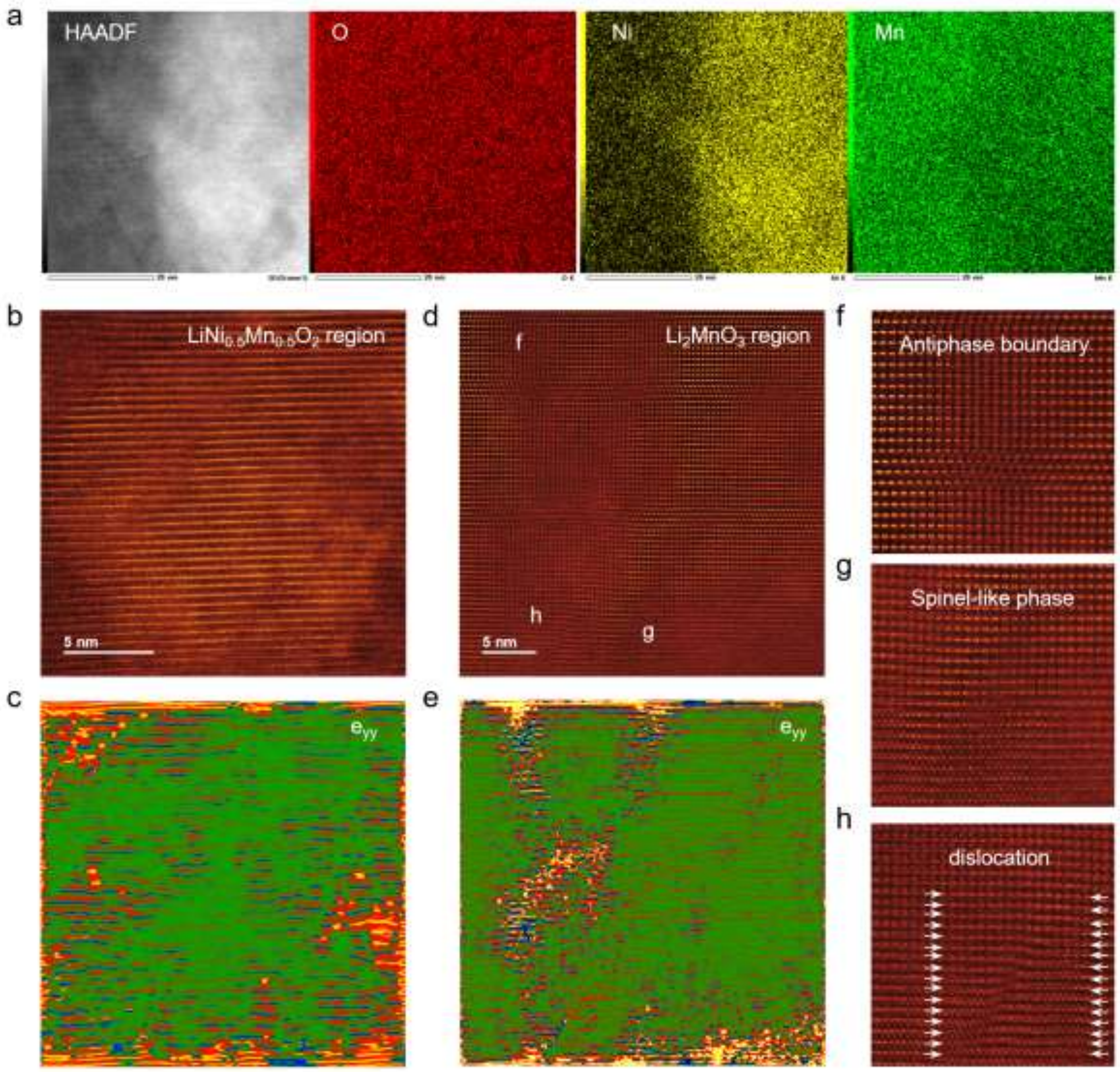

Figure S21. (a) STEM-EDS mappings for LMR-900 ℃ cathode at charged state showing the boundary between two phases. (b) High resolution HAADF-STEM image and (c) corresponding GPA analysis showing the distorted structure of $LiNi_{0.5}Mn_{0.5}O_2$ region at charged state. (d) High resolution HAADF-STEM image and (e) corresponding GPA analysis showing the distorted structure of Li_2MnO_3 at charged state. (f-h) Enlarged HAADF images extracted from the marked position in (d) showing the emerged defects after delithiation.

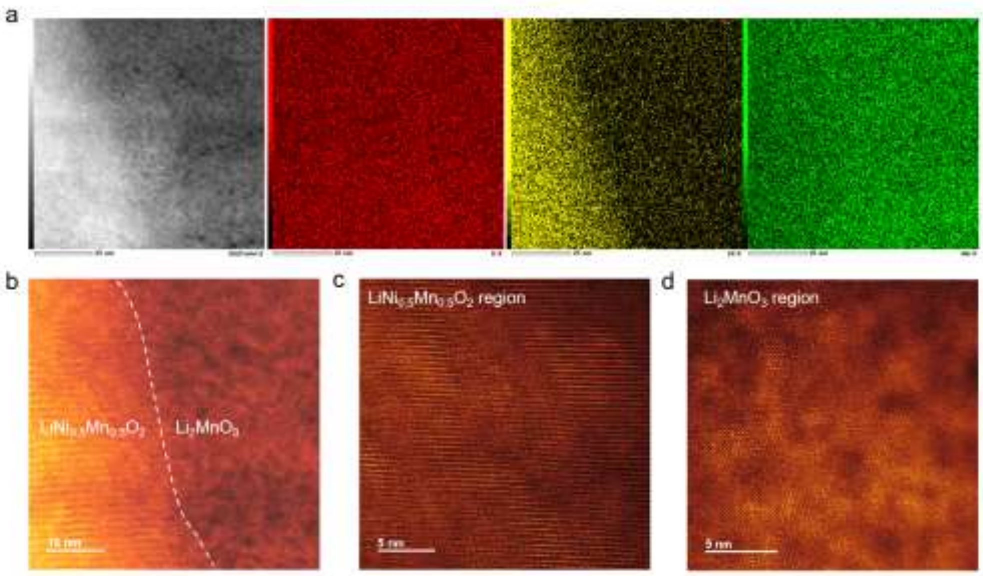

Figure S22. (a) STEM-EDS mappings for LMR-900 ℃ cathode at discharged state after 10 cycles showing the boundary between two phases. (b-d) High resolution HAADF-STEM image for the interphase region, $LiNi_{0.5}Mn_{0.5}O_2$ region and Li_2MnO_3 region after cycling.

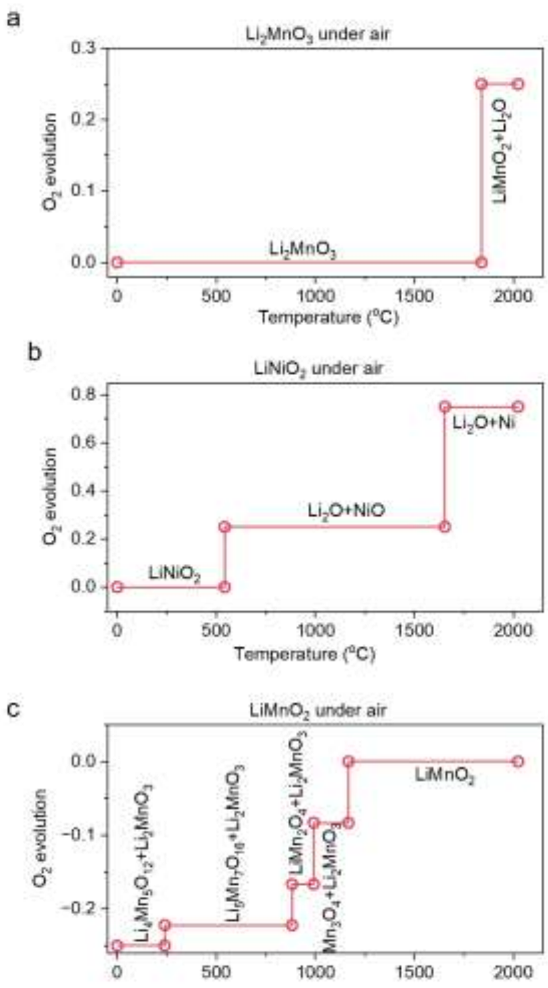

Figure S23. The calculated stable phases under different temperature for (a) Li_2MnO_3, (b) $LiNiO_2$ and (c) $LiMnO_2$, respectively.

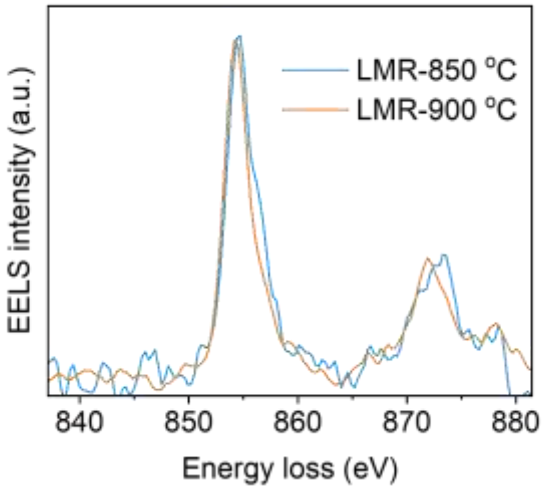

Figure S24. The EELS spectra comparison of LMR-850 °C and LMR-900 °C.

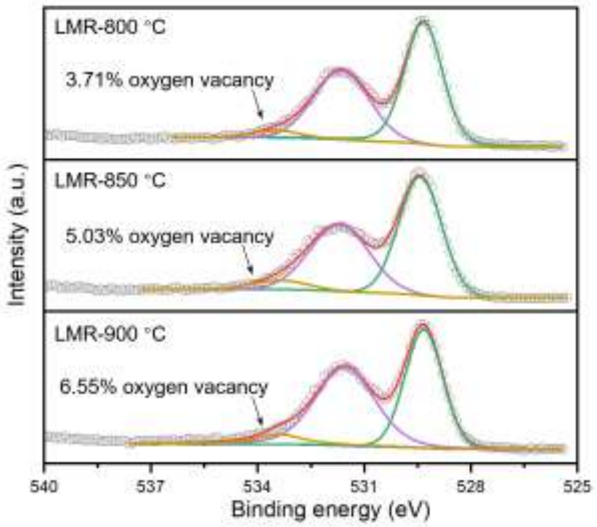

Figure S25. XPS results for LMR cathodes synthesized under different temperatures. The fitting results and corresponding oxygen vacancy content are also indicated with the spectra.
